# Supplementary material for: Selective interactions at pre-replication complexes categorize baseline and dormant origins
Source: Nat Commun. 2025 May 3;16:4140. doi: 10.1038/s41467-025-59509-4 (PMC12049448; doi:10.1038/s41467-025-59509-4)
Supplement: Supplementary file 1 — Supplementary Information [file 41467_2025_59509_MOESM1_ESM.pdf]

Supplementary Information for

Selective interactions at pre-replication complexes categorize baseline and dormant origins

Bhushan L. Thakur[1], Christophe E. Redon[1], Haiqing Fu[1], Robin Sebastian[1], Nana A. Kusi[1], Sophie Z. Zhuang[1], Lorinc S. Pongor[1,2], Vilhelm A Bohr[3] and Mirit I. Aladjem[1,*]

Affiliations:

1 Developmental Therapeutics Branch, Center for Cancer Research, NCI, NIH.

2 Current address: Cancer Genomics and Epigenetics Research Group, HCEMM, Szeged, Hungary

3 Department of ICMM, University of Copenhagen, Denmark

* Corresponding author: aladjemm@mail.nih.gov

The PDF file includes:

Supplemental Figures 1 to 5 and legends.

Supplementary figure 1

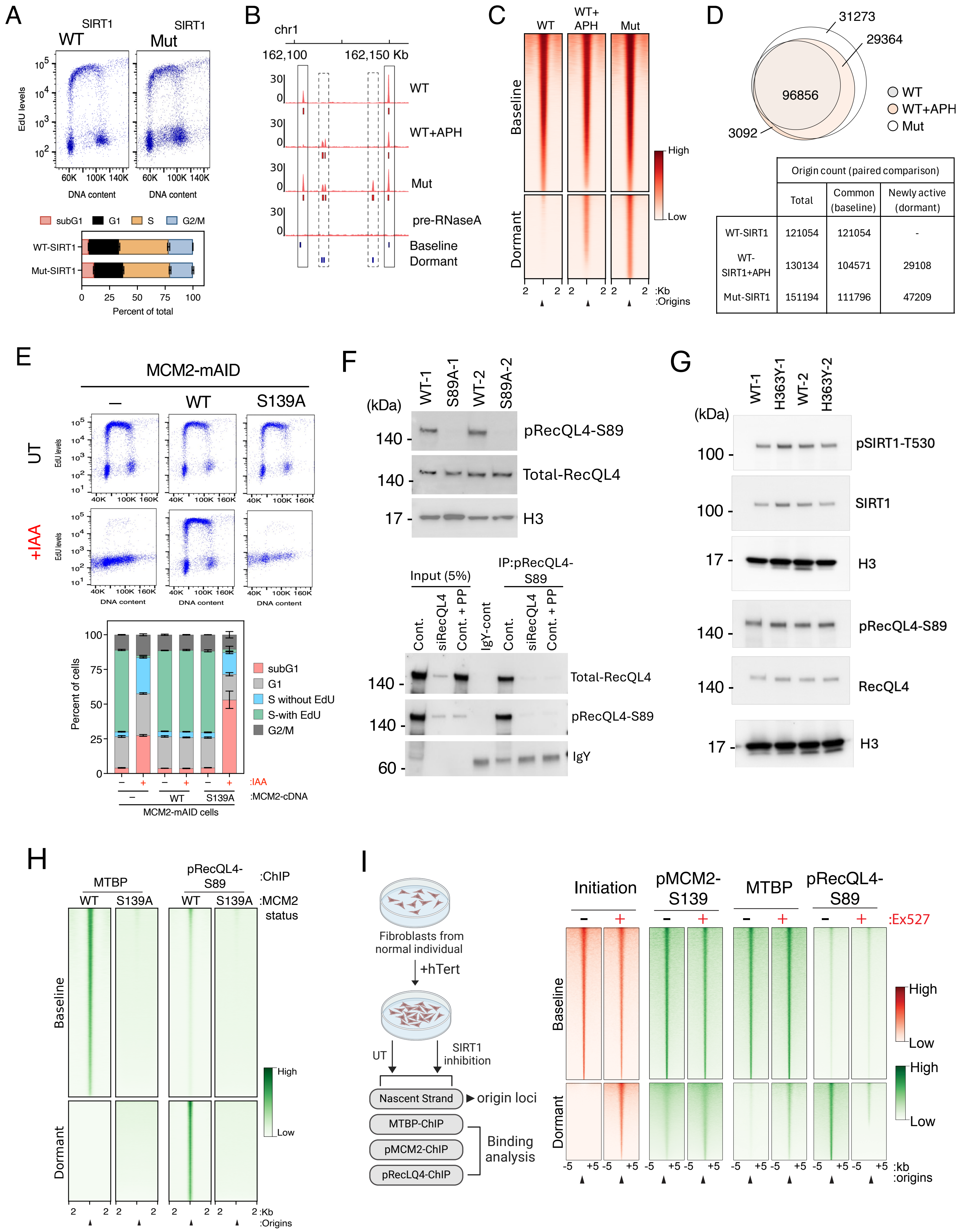

	Origin count (paired comparison)		
	Total	Common (baseline)	Newly active (dormant)
WT-SIRT1	121054	121054	-
WT-SIRT1+APH	130134	104571	29108
Mut-SIRT1	151194	111796	47209

Supplementary Figure 1: **Characterization of MCM2 and SIRT1 Models in HCT116, and Impact of SIRT1 Inhibition on Fibroblasts.**

A: Top, representative cell cycle distribution of WT^{SIRT1} and Mut^{SIRT1} cells, measured by flow cytometry using an EdU incorporation/DAPI assay. See source data 1 for the gating strategy. Bottom, quantification of cell cycle phases for WT^{SIRT1} (WT, n=4) and Mut^{SIRT1} (Mut, n=4). Stacked bars show the mean, error bars indicate standard deviation (SD).

B: A genome browser snapshot of origin mapping by nascent strand sequencing at the genomic region depicted in figure 1A. From the top, coverage tracks of nascent strands from untreated WT^{SIRT1} (WT), aphidicolin treated WT^{SIRT1} (WT+APH) and untreated Mut^{SIRT1} (Mut) cells and an pre-RNAseA-treated control. MACS peaks corresponding to baseline (solid black boxes) and dormant origins (dotted black boxes) locations are shown below the sequencing tracks.

C: A heatmap illustrating nascent strand abundance at baseline and dormant replication origins in HCT116 cells harboring WT^{SIRT1} (WT), WT^{SIRT1} treated with APH (10mM for 1hr, WT+APH) and Mut^{SIRT1} (Mut).

D. Top, a Venn diagram depicting the colocalization of replication initiation sites in the samples shown in Panel C. Bottom, a table showing identified replication origins (data from two replicates).

E: Top, a cell cycle distribution of mAID-MCM2 containing HCT116 cells complemented with either MCM2-WT or MCM2-S139A with and without exposure to IAA (A). The expression levels of mutant proteins were similar to those of the wild-type, and both were comparable to the endogenous protein. Bottom, quantification of cell cycle phases for MCM2-mAID (Cont, n=3, IAA, n=4), WT-MCM2 (Cont, n=3, IAA, n=4) and S139A-MCM2 (Cont, n=3, IAA, n=4). Stacked bars show the mean, error bars indicate SD.

F: Characterization of the custom-made pRecQL4 antibody used in the study. Top, whole cell fractions of two independently isolated clones of HCT116 cells harboring intact RecQL4 (WT a and 2) or HCT116 cells harboring RecQL4-S89A (S89A 1 and 2) immunoblotted using the pRecQL4-S89, total-RecQL4 and histone H3 antibodies, showing the specificity of pRecQL4-S89 antibody. Bottom, chromatin fractions of HCT116 cells harboring intact RecQL4 (WT) or HCT116 cells treated with siRNA targeting RecQL4 (siRecQL4), and control lysates treated with

lambda protein phosphatase (PP) were immunoprecipitated using the pRecQL4-S89 antibodies, followed by immunoblotting with total-RecQL4 and pRecQL4-S89 antibodies.

G: Abundance of phosphorylated SIRT1-T530, total SIRT1, pRecQL4-S89 and RecQL4 in whole cell exctracts of the cells harboring WT and mutant (H363Y) SIRT1. The expression levels of mutant proteins were similar to those of the wild-type proteins, and both were comparable to the endogenous protein.

H: ChIP-seq using antibodies directed against MTBP and pRecQL4-S89 in cells harboring MCM2-WT and MCM2-S139A derived and exposed to IAA (500μM for 16hr) according to the experimental strategy shown in Figure 1C. Binding sites are stratified for baseline and dormant origins as shown in Figure 1, A and B.

I: Left, experimental procedure. Normal human fibroblasts (see Methods for details) were immortalized and dormant origins were activated by SIRT1 inhibition (Ex527 1 mM every 24hr for 3 days). Right, a heatmap illustrating the strength of origin activation (in red) and the binding of pMCM2-S139, MTBP and pRecQL4-S89 (in green) at baseline and dormant replication origin sites in untreated and EX527-treated fibroblasts.

Supplementary figure 2

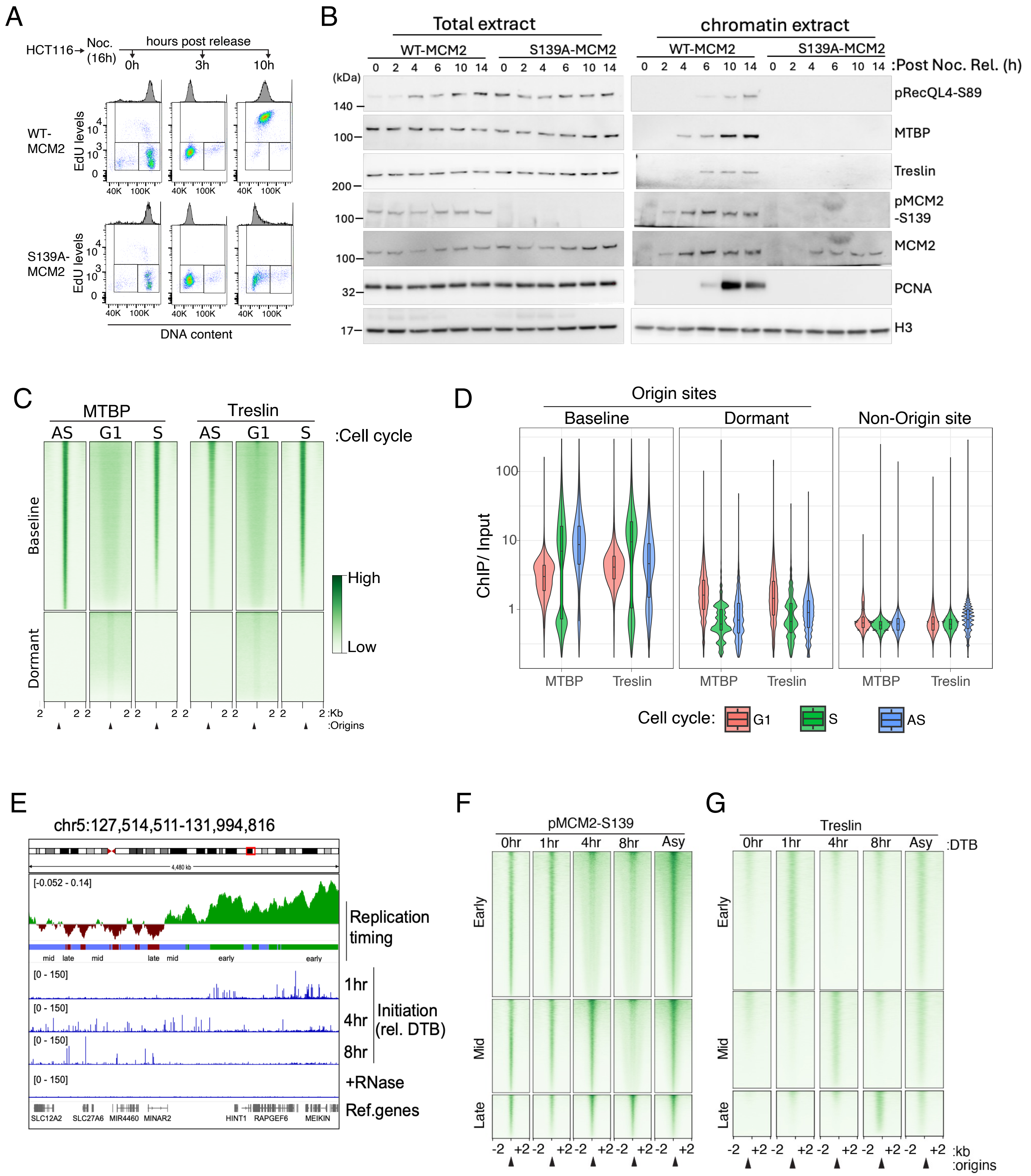

Supplementary Figure 2: Cell cycle specific binding of MTBP-Treslin, and pMCM2-S139. A: Cells harboring WT-MCM2 and S139A-MCM2 were exposed to nocodazole (noc.) along with IAA (500μM for 16hr) and released for the indicated time period. Cell cycle distributions were analysed by flow cytometry using an EdU incorporation/DAPI assay. Top represents DNA content (grey).

B: An immunoblot measuring the abundance of pRecQL4, MTBP, Treslin, pMCM2-S139, MCM2 and PCNA in the whole cell and chromatin extracts of HCT116 cells harboring WT-MCM2 and S139A-MCM2 treated as shown in panel A. Representative of three independent replicates.

C: Heatmaps depicting the distribution of MTBP and Treslin binding sites centered on baseline and dormant origins at the indicated timepoint after release from a nocodazole-induced G2/M block as described in the legend to panel A. As = Asynchronous, G1 = 3 hr and S = 10 hr post release from noc. Baseline and dormant origins were stratified as described in Figure 1, A and B.

D: Quantification of the extent of origin binding of MTBP and Treslin to baseline and dormant origins and to non-origin regions following a nocodazole-induced G2/M block (data correspond to the heatmap shown in panel C). AS = Asynchronous, G1 = 3hr and S = 10hr release from noc.

E: A genome browser screenshot of a genomic region residing on Chromosome 5. From top, replication timing profile in HCT116 cells with annotated regions (early, mid-, and late-replicating). NS-seq from DTB-released samples at the 0 hr, 1 hr, 4 hr, and 8 hr timepoints; an RNaseA pretreated (1hr) control.

F & G: ChIP-seq was performed at the same (DTB-released) time points as in figure 2B to map interactions of baseline origins with pMCM2-S139 (F) and Treslin (G). The heatmaps illustrate signal strength on early, mid, and late origins, with signal intensities centered on origins as stratified in figure 2C.

Supplementary figure 3

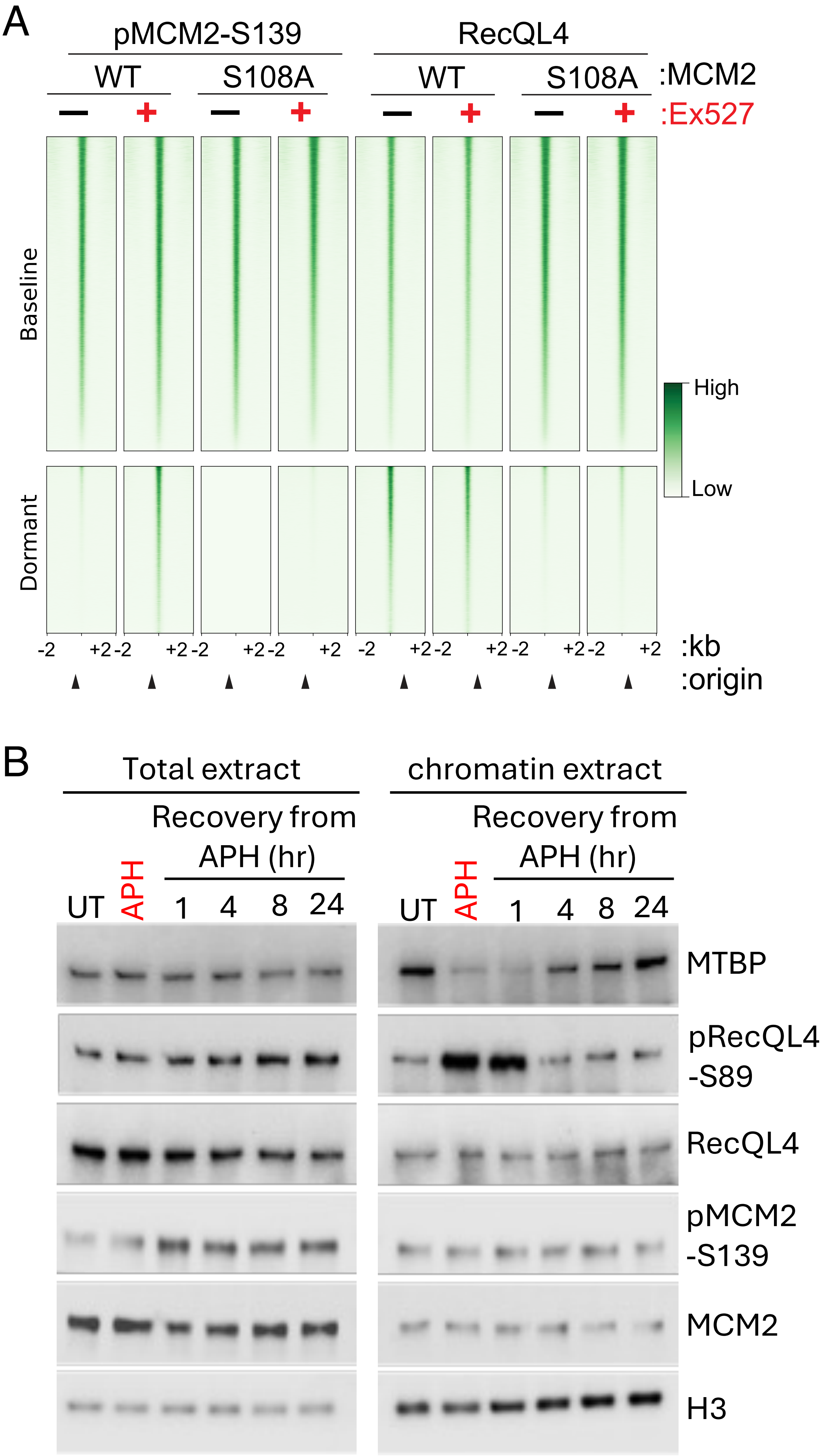

Supplementary Figure 3: Binding of pMCM2-S139/RecQL4 in MCM2-S108A cells and MTBP/pRecQL4 during replication stress recovery.

A: HCT116 cells harboring MCM2-WT and MCM2-S108A were generated as illustrated in Figure 1C. Heatmaps are showing the binding of pMCM2-S139 and total RecQL4 to baseline and dormant origins without (UT) and with exposure to Ex527 (1 mM every 24hr for 2 days).

B: An immunoblot showing the abundance of the indicated proteins in total and chromatin extracts from HCT116 cells untreated, treated and released from exposure to APH (10 μM for 1hr).

Supplementary figure 4

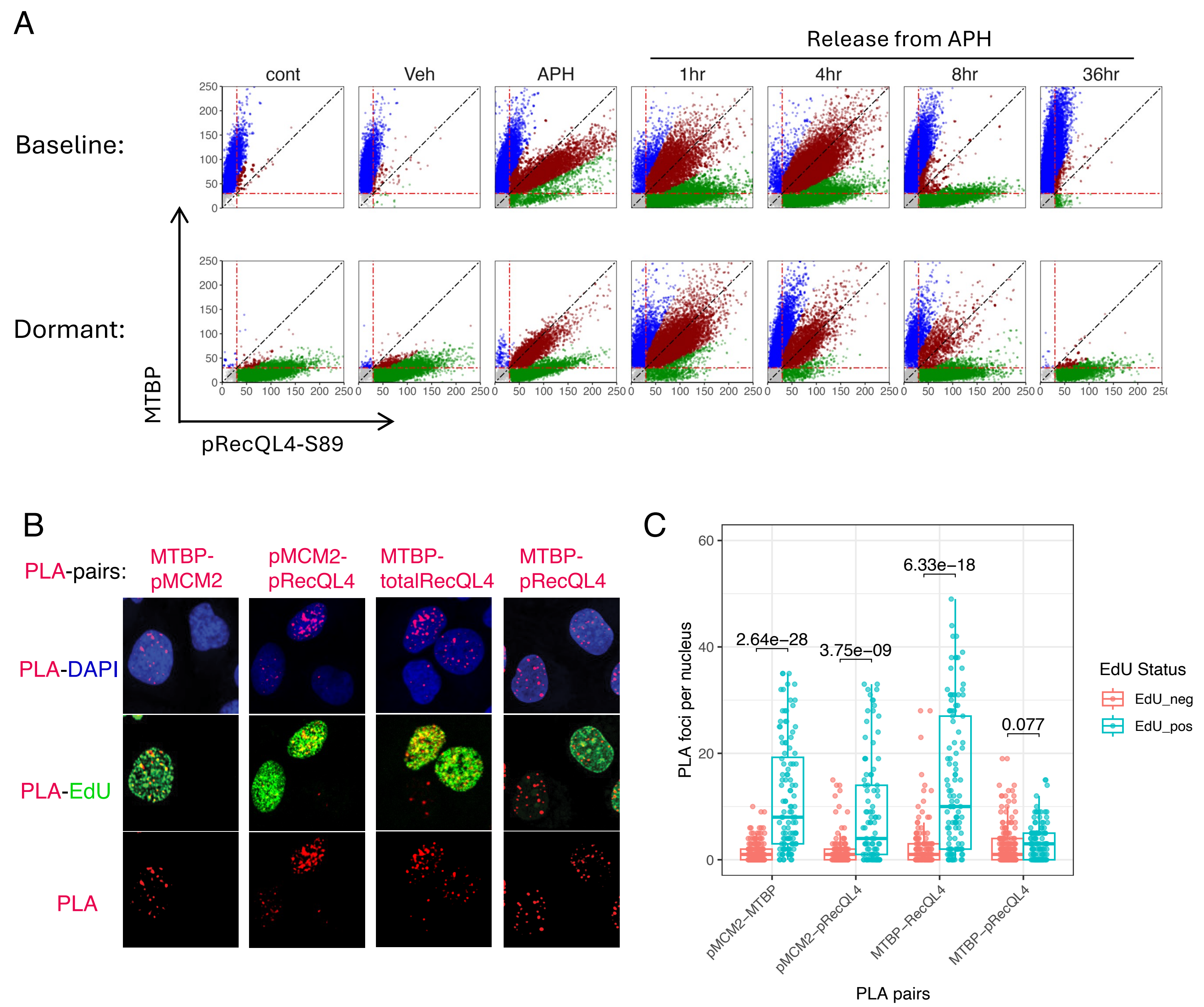

Supplementary Figure 4: MTBP and pRecQL4 association with replication origins during replication stress recovery and interaction of pMCM2-S139, MTBP, and pRecQL4 in replicating and non-replicating cells.

A: Binding of MTBP and pRecQL4-S89 to dormant origins in APH treated HCT116 cells collected at the time points indicated in Figure 4A. In this plot, each origin is represented by a single dot, the Y-axis represents the strength of MTBP binding and the X-axis represents the strength of RecQL4 binding.

B and C: A proximity-ligation assay detects interactions of pMCM2-S139 with MTBP and pRecQL4-S89, or MTBP with total-RecQL4 and pRecQL4-S89 in HCT116 cells, with EdU used to identify S-phase and non-replicating cells. B, representative images. C, PLA spot quantification for each condition, with sub-grouping by EdU positivity. EdU_pos =cells with EdU signal, EdU_neg= cells without EdU signal. The box-whisker plot shows quantification PLA pair of 2 biological replicates: pMCM2-MTBP (n= 298), pMCM2-pRecQL4 (n= 239), MTBP-RecQL4 (n=252) and MTBP-pRecQL4 (n= 313). Boxplot shows 25th percentile, median and 75th percentile. Statistical analysis was performed for the comparison of EdU-negative and EdU-positive cells using a two-sided unpaired Wilcox text, exact p-values are shown in the plot.

Supplementary figure 5

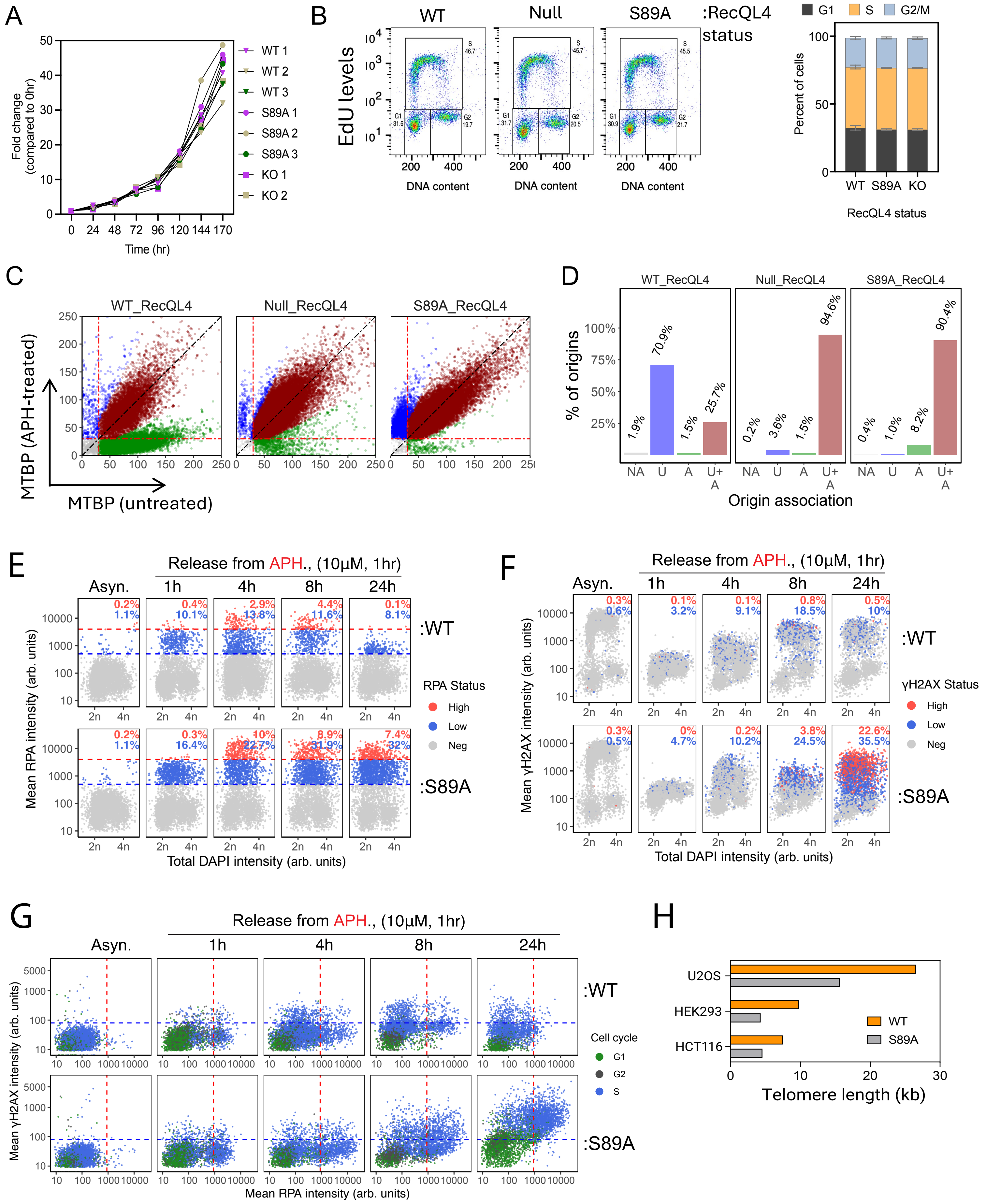

Supplementary Figure 5: Phospho-RecQL4 promotes efficient recovery from replication stress.

A: Growth curves for HCT116 clones depleted of RecQL4 (Null) or RecQL4 depleted cells harboring RecQL4-WT (WT) and RecQL4-S89A (S89A). 1, 2, and 3 indicates individual stable clones.

B: Left, representative cell cycle distribution of clone 2 for HCT116 cells without RecQL4 (Null) or harboring RecQL4-WT and RecQL4-S89A. Right, quantification of the clones of HCT116 cells without RecQL4 (Null) or harboring RecQL4-WT and RecQL4-S89A as shown in panel A. Quantification of cell cycle phases for WT (n=2), S89A (n=2) and KO (n=2). Stacked bars show the mean, error bars indicate SD.

C: Comparison of binding of MTBP to replication origins between untreated and with APH-treated HCT116 cells without RecQL4 (Null) or harboring RecQL4-WT and RecQL4-S89A, see main figure 5B for heatmap representation.

D: Bar plots indicating the fractions (percent) of baseline origins from each shaded area of the XY-plots shown in panel C. The plots indicate the fractions (percent) of baseline origins for subgroups: NA, no association; U, associated only in untreated cells; A, associated only in APH-treaed cells, U+A, associated with origins in untreated and APH-treated cells.

E: QIBC profiles categorizing RPA signals (y-axis) as high, low, and negative in HCT116 cells during recovery from APH. X-axis, DNA content measured by DAPI intensity. Blue and red dotted lines represent thresholds for low and high-RPA signals.

F: Quantification of the recovery of HCT116 cells after exposure to APH. Cells with phospho-proficient (top panels) and phospho-deficient (lower panel) RecQL4 were labeled with EdU and γH2AX. EdU incorporation (y-axis) was plotted versus DAPI intensity (x-axis). High- and low-γH2AX positive cells are shown in red and blue, respectively.

G: A density scatter plot of RPA versus γH2AX signals measured during the recovery of HCT116 cells with phospho-proficient (WT) and phospho-deficient (S89A) RecQL4 from exposure to APH. The cells are color-coded to show cell cycle phases.

H: Analysis of telomere length from whole genome sequencing data of indicated cell lines using TelSeq [42]. A barplot showing telomere length from WT (n=2) and S89A (n=2) for each cell line.
