# Supplementary material for: Selective interactions at pre-replication complexes categorize baseline and dormant origins
Source: Nat Commun. 2025 May 3;16:4140. doi: 10.1038/s41467-025-59509-4 (PMC12049448; doi:10.1038/s41467-025-59509-4)
Supplement: Supplementary file 2 — Reporting Summary [file 41467_2025_59509_MOESM2_ESM.pdf]

nature portfolio

Corresponding author(s): Mirit I. Aladjem

Last updated by author(s): Apr 6, 2025

Reporting Summary

Nature Portfolio wishes to improve the reproducibility of the work that we publish. This form provides structure for consistency and transparency in reporting. For further information on Nature Portfolio policies, see our Editorial Policies and the Editorial Policy Checklist.

Statistics

For all statistical analyses, confirm that the following items are present in the figure legend, table legend, main text, or Methods section.

n/a	Confirmed	
☐	☒	The exact sample size (*n*) for each experimental group/condition, given as a discrete number and unit of measurement
☐	☒	A statement on whether measurements were taken from distinct samples or whether the same sample was measured repeatedly
☐	☒	The statistical test(s) used AND whether they are one- or two-sided<br>*Only common tests should be described solely by name; describe more complex techniques in the Methods section.*
☒	☐	A description of all covariates tested
☒	☐	A description of any assumptions or corrections, such as tests of normality and adjustment for multiple comparisons
☐	☒	A full description of the statistical parameters including central tendency (e.g. means) or other basic estimates (e.g. regression coefficient) AND variation (e.g. standard deviation) or associated estimates of uncertainty (e.g. confidence intervals)
☐	☒	For null hypothesis testing, the test statistic (e.g. *F*, *t*, *r*) with confidence intervals, effect sizes, degrees of freedom and *P* value noted<br>*Give P values as exact values whenever suitable.*
☒	☐	For Bayesian analysis, information on the choice of priors and Markov chain Monte Carlo settings
☒	☐	For hierarchical and complex designs, identification of the appropriate level for tests and full reporting of outcomes
☐	☒	Estimates of effect sizes (e.g. Cohen's *d*, Pearson's *r*), indicating how they were calculated

Our web collection on statistics for biologists contains articles on many of the points above.

Software and code

Policy information about availability of computer code

Data collection

1. Flow cytometry data were collected using a BD LSR Fortessa cell analyzer with FACSDiva software (v6.2).
2. Nascent-seq data were obtained with an Illumina Genome Analyzer II (Solexa) using 150 bp paired-end sequencing.
3. ChIP-seq data were generated with an Illumina NextSeq using 75 bp single-end sequencing.
4. Microscopy images were captured using a Yokogawa CV7000 confocal spinning disk microscope (60X water immersion lens, NA 1.2).
5. SpectraMax i3x reader and the SoftMax Pro 7 software (Molecular Devices)

Data analysis

1, Flow cytometry data were analyzed with FlowJo 10.6.
2, Nascent-seq and ChIP-seq data were processed using standard software and pipelines, including FastQC (v0.11.5), Trimmomatic (version 0.36) and Trim Galore (version 0.4.5); FastQC (version 0.11.5) [https://www.bioinformatics.babraham.ac.uk/projects/fastqc/], bwa aligner (version 0.7.17);MACS2 (version 2.1.1.20160309); MACS2 metric in R (version 3.5.1); R scripts (https://github.com/ncbi/BAMscale/wiki); samtools (v1.21); bedtools (2.31.1) were used to generate BAM, BigWig, and peak files (broadPeak and narrowPeak).
3, Data visualization was done using DeepTools (v3.5.0), PlotHeatmap (v3.5.0), R scripts (https://github.com/ncbi/BAMscale/wiki), and IGV 2.11.
4, Graphs and statistical analysis were created with R or GraphPad Prism 10.0.0.
5, Violin plots for sequencing data were made with ggplot (v3.4.3) in R.

All the data analysis performed using standard pipelines using publicly available softwares and tools.

For manuscripts utilizing custom algorithms or software that are central to the research but not yet described in published literature, software must be made available to editors and reviewers. We strongly encourage code deposition in a community repository (e.g. GitHub). See the Nature Portfolio guidelines for submitting code & software for further information.

Data

Policy information about availability of data

All manuscripts must include a data availability statement. This statement should provide the following information, where applicable:

- Accession codes, unique identifiers, or web links for publicly available datasets
- A description of any restrictions on data availability
- For clinical datasets or third party data, please ensure that the statement adheres to our policy

ChIP and NS sequencing data for HCT116 cells were deposited in GEO (GSE276856) with reviewers token gfmdcwayftobpep. Fibroblast sequencing and replication timing data are publicly available in GEO under accessions GSE247469 and GSE172417, respectively.

Research involving human participants, their data, or biological material

Policy information about studies with human participants or human data. See also policy information about sex, gender (identity/presentation), and sexual orientation and race, ethnicity and racism.

Reporting on sex and gender	NA
Reporting on race, ethnicity, or other socially relevant groupings	NA
Population characteristics	NA
Recruitment	NA
Ethics oversight	NA

Note that full information on the approval of the study protocol must also be provided in the manuscript.

Field-specific reporting

Please select the one below that is the best fit for your research. If you are not sure, read the appropriate sections before making your selection.

☒ Life sciences ☐ Behavioural & social sciences ☐ Ecological, evolutionary & environmental sciences

For a reference copy of the document with all sections, see nature.com/documents/nr-reporting-summary-flat.pdf

Life sciences study design

All studies must disclose on these points even when the disclosure is negative.

Sample size	For microscopy cell counting, we analyze 1000-5000 cells for each replicates (3 to 4 biological replicates) that gave sufficient statistics for the effect sizes of interest.
Data exclusions	No data were excluded from analysis.
Replication	For all the experiments, we did at least three independent biological replicates except for sequencing experiments, which have at least 2 independent biological replicates. Results were consistently replicated across multiple experiments with all replicates generating similar results.
Randomization	Microscopy images were collected using a scanning microscope from 9 fields per well, with 3 wells per replicate, ensuring sufficient and unbiased randomization. All cells that were imaged included in the analysis. Randomization was not applied to other experiments.
Blinding	Blinding is also not necessary because the results are quantitative and did not require subjective judgment or interpretation.

Reporting for specific materials, systems and methods

We require information from authors about some types of materials, experimental systems and methods used in many studies. Here, indicate whether each material, system or method listed is relevant to your study. If you are not sure if a list item applies to your research, read the appropriate section before selecting a response.

Materials & experimental systems

n/a	Involved in the study	
☐	☒	Antibodies
☐	☒	Eukaryotic cell lines
☒	☐	Palaeontology and archaeology
☒	☐	Animals and other organisms
☒	☐	Clinical data
☒	☐	Dual use research of concern
☒	☐	Plants

Methods

n/a	Involved in the study	
☐	☒	ChIP-seq
☐	☒	Flow cytometry
☒	☐	MRI-based neuroimaging

Antibodies

Antibodies used

The primary antibodies used were RecQL4 (Cell Signaling, 2814), MCM2 (Cell Signaling, 12079), phospho-MCM2 (S139) (Cell Signaling, 12958), MTBP (Novus biologicals, NBP1-86408),PCNA (Millipore cat# MAB424R) RPA2 (Millipore cat# MABE285), gamma-H2AX (Millipore cat# 05-636) and histone H3 (Millipore, 07-690). Antibodies against pSIRT1 T530 (25) and pRecQL4 S89 (this work, see fig S1C for validation) were custom-made. Anti-Rb and anti-MS HRP-labeled antibodies (Cell Signaling, 7074 and 7076) were used as secondary antibodies for western blotting.
Secondary antibodies:
Alexa 488 conjugated anti-mouse IgG, Alexa 488 conjugated anti-rabbit IgG and Alexa 568 conjugated anti-rabbit IgG (Thermo Fisher Scientific, A11029, A11008 and A21428), Anti-Rb and anti-MS HRP-labeled antibodies (Cell Signaling, cat# 7074 and cat# 7076).

Validation

All antibodies were validated using immunoblots based on the molecular weight of the target. MCM2, phospho-MCM2 (S139), MTBP GammaH2AX, RPA, PCNA, Histone H3 and Phospho-Chk1 antibodies also have been reported in many publications. pRecQL4 S89 is reported in this work, see fig S1F for validation.

Eukaryotic cell lines

Policy information about cell lines and Sex and Gender in Research

Cell line source(s)

HCT116 (CCL247) and U2OS (HTB96) cell lines, both are from ATCC. Fibroblasts lines AG04446 collected from a healthy individual donor at age 48 were obtained from the Coriell Institute's Aging Cell Depository.

Authentication

Stable clones were validated by western blot

Mycoplasma contamination

Mycoplasma tested negative with both cell lines.

Commonly misidentified lines
(See ICLAC register)

No commonly misidentified cell lines were used.

Plants

Seed stocks

NA

Novel plant genotypes

NA

Authentication

NA

ChIP-seq

Data deposition

☒ Confirm that both raw and final processed data have been deposited in a public database such as GEO.

☒ Confirm that you have deposited or provided access to graph files (e.g. BED files) for the called peaks.

Data access links
May remain private before publication.

The ChIP- and NS-sequencing raw and processed data generated in this study have been deposited in the GEO under accession number GSE276856 [https://www.ncbi.nlm.nih.gov/geo/query/acc.cgi?acc=GSE276856], GSE247469 [https://www.ncbi.nlm.nih.gov/geo/query/acc.cgi?acc=GSE247469] and GSE172417 [https://www.ncbi.nlm.nih.gov/geo/query/acc.cgi?acc=GSE172417] and are publicly available.

Files in database submission

BAM files:
APH_rec_1hr_MTBP_rep1.sorted.dedup.bam
APH_rec_1hr_MTBP_rep2.sorted.dedup.bam
APH_rec_1hr_pRQ4_rep1.sorted.dedup.bam
APH_rec_1hr_pRQ4_rep2.sorted.dedup.bam
APH_rec_4hr_MTBP_rep1.sorted.dedup.bam
APH_rec_4hr_MTBP_rep2.sorted.dedup.bam
APH_rec_4hr_pRQ4_rep1.sorted.dedup.bam
APH_rec_4hr_pRQ4_rep2.sorted.dedup.bam
APH_rec_8hr_MTBP_rep1.sorted.dedup.bam
APH_rec_8hr_MTBP_rep2.sorted.dedup.bam
APH_rec_8hr_pRQ4_rep1.sorted.dedup.bam
APH_rec_8hr_pRQ4_rep2.sorted.dedup.bam
AS_MTBP_rep1.sorted.dedup.bam
AS_MTBP_rep2.sorted.dedup.bam
AS_pMCM2_rep1.sorted.dedup.bam
AS_pMCM2_rep2.sorted.dedup.bam
AS_pRQ4_rep1.sorted.dedup.bam
AS_pRQ4_rep2.sorted.dedup.bam
DTB_0hr_MTBP_rep1.sorted.dedup.bam
DTB_0hr_MTBP_rep2.sorted.dedup.bam
DTB_0hr_pMCM2_rep1.sorted.dedup.bam
DTB_0hr_pMCM2_rep2.sorted.dedup.bam
DTB_0hr_pRQ4_rep1.sorted.dedup.bam
DTB_0hr_pRQ4_rep2.sorted.dedup.bam
DTB_1hr_MTBP_rep1.sorted.dedup.bam
DTB_1hr_MTBP_rep2.sorted.dedup.bam
DTB_1hr_pMCM2_rep1.sorted.dedup.bam
DTB_1hr_pMCM2_rep2.sorted.dedup.bam
DTB_1hr_pRQ4_rep1.sorted.dedup.bam
DTB_1hr_pRQ4_rep2.sorted.dedup.bam
DTB_4hr_MTBP_rep1.sorted.dedup.bam
DTB_4hr_MTBP_rep2.sorted.dedup.bam
DTB_4hr_pMCM2_rep1.sorted.dedup.bam
DTB_4hr_pMCM2_rep2.sorted.dedup.bam
DTB_4hr_pRQ4_rep1.sorted.dedup.bam
DTB_4hr_pRQ4_rep2.sorted.dedup.bam
DTB_8hr_MTBP_rep1.sorted.dedup.bam
DTB_8hr_MTBP_rep2.sorted.dedup.bam
DTB_8hr_pMCM2_rep1.sorted.dedup.bam
DTB_8hr_pMCM2_rep2.sorted.dedup.bam
DTB_8hr_pRQ4_rep1.sorted.dedup.bam
DTB_8hr_pRQ4_rep2.sorted.dedup.bam
DTB_Input_1hr_R1_001.sorted.dedup.bam
DTB_Input_4hr_R1_001.sorted.dedup.bam
DTB_Input_8hr_R1_001.sorted.dedup.bam
H3Y_MTBP_rep1.sorted.dedup.bam
H3Y_MTBP_rep2.sorted.dedup.bam
H3Y_pMCM2_rep1.sorted.dedup.bam
H3Y_pMCM2_rep2.sorted.dedup.bam
H3Y_pRQ4_rep1.sorted.dedup.bam
H3Y_pRQ4_rep2.sorted.dedup.bam
H3Y_RQ4_rep1.sorted.dedup.bam
H3Y_RQ4_rep2.sorted.dedup.bam
Input_AS_R1_001.sorted.dedup.bam
Input_H3Y_R1_001.sorted.dedup.bam
Input_S89A_RQ_R1_001.sorted.dedup.bam
Input_UT_1_R1_001.sorted.dedup.bam
Input_UT_2_R1_001.sorted.dedup.bam
Input_WT_APH_R1_001.sorted.dedup.bam
Input_WT_M2.sorted.dedup.bam
Input_WT_R1_001.sorted.dedup.bam
Input_WT_RQ_R1_001.sorted.dedup.bam
MCM2_WT_Ex_MTBP_rep1.sorted.dedup.bam
MCM2_WT_Ex_MTBP_rep2.sorted.dedup.bam
MCM2_WT_Ex_pRQ4_rep1.sorted.dedup.bam
MCM2_WT_Ex_pRQ4_rep2.sorted.dedup.bam
MCM2_WT_MTBP_rep1.sorted.dedup.bam
MCM2_WT_MTBP_rep2.sorted.dedup.bam
MCM2_WT_pRQ4_rep1.sorted.dedup.bam
MCM2_WT_pRQ4_rep2.sorted.dedup.bam
RQ4_null_MTBP_APH_rep1.sorted.dedup.bam
RQ4_null_MTBP_APH_rep2.sorted.dedup.bam
RQ4_null_MTBP_UT_rep1.sorted.dedup.bam
RQ4_null_MTBP_UT_rep2.sorted.dedup.bam

RQ4_null_pRQ4_APH_rep1.sorted.dedup.bam
RQ4_null_pRQ4_APH_rep2.sorted.dedup.bam
RQ4_null_pRQ4_UT_rep1.sorted.dedup.bam
RQ4_null_pRQ4_UT_rep2.sorted.dedup.bam
RQ4_WT_MTBP_APH_rep1.sorted.dedup.bam
RQ4_WT_MTBP_APH_rep2.sorted.dedup.bam
RQ4_WT_MTBP_UT_rep1.sorted.dedup.bam
RQ4_WT_MTBP_UT_rep2.sorted.dedup.bam
RQ4_WT_pRQ4_APH_rep1.sorted.dedup.bam
RQ4_WT_pRQ4_APH_rep2.sorted.dedup.bam
RQ4_WT_pRQ4_UT_rep1.sorted.dedup.bam
RQ4_WT_pRQ4_UT_rep2.sorted.dedup.bam
S108A_Ex_MTBP_rep1.sorted.dedup.bam
S108A_Ex_MTBP_rep2.sorted.dedup.bam
S108A_Ex_pRQ4_rep1.sorted.dedup.bam
S108A_Ex_pRQ4_rep2.sorted.dedup.bam
S108A_MTBP_rep1.sorted.dedup.bam
S108A_MTBP_rep2.sorted.dedup.bam
S108A_pRQ4_rep1.sorted.dedup.bam
S108A_pRQ4_rep2.sorted.dedup.bam
S89A_MTBP_APH_rep1.sorted.dedup.bam
S89A_MTBP_APH_rep2.sorted.dedup.bam
S89A_MTBP_UT_rep1.sorted.dedup.bam
S89A_MTBP_UT_rep2.sorted.dedup.bam
S89A_pRQ4_APH_rep1.sorted.dedup.bam
S89A_pRQ4_APH_rep2.sorted.dedup.bam
S89A_pRQ4_UT_rep1.sorted.dedup.bam
S89A_pRQ4_UT_rep2.sorted.dedup.bam
UT_MTBP_rep1.sorted.dedup.bam
UT_MTBP_rep2.sorted.dedup.bam
UT_pRQ4_rep1.sorted.dedup.bam
UT_pRQ4_rep2.sorted.dedup.bam
WT_APH_MTBP_rep1.sorted.dedup.bam
WT_APH_MTBP_rep2.sorted.dedup.bam
WT_APH_pMCM2_rep1.sorted.dedup.bam
WT_APH_pMCM2_rep2.sorted.dedup.bam
WT_APH_pRQ4_rep1.sorted.dedup.bam
WT_APH_pRQ4_rep2.sorted.dedup.bam
WT_APH_RQ4_rep1.sorted.dedup.bam
WT_APH_RQ4_rep2.sorted.dedup.bam
WT_MTBP_rep1.sorted.dedup.bam
WT_MTBP_rep2.sorted.dedup.bam
WT_pMCM2_rep1.sorted.dedup.bam
WT_pMCM2_rep2.sorted.dedup.bam
WT_pRQ4_rep1.sorted.dedup.bam
WT_pRQ4_rep2.sorted.dedup.bam
WT_RQ4_rep1.sorted.dedup.bam
WT_RQ4_rep2.sorted.dedup.bam
NS_H3Y_S1.sorted.dedup.bam
NS_H3Y_S2.sorted.dedup.bam
NS_WT_0hr_S1.sorted.dedup.bam
NS_WT_0hr_S2.sorted.dedup.bam
NS_WT_1hr_S1.sorted.dedup.bam
NS_WT_1hr_S2.sorted.dedup.bam
NS_WT_4hr_S1.sorted.dedup.bam
NS_WT_4hr_S2.sorted.dedup.bam
NS_WT_7hr_S1.sorted.dedup.bam
NS_WT_7hr_S2.sorted.dedup.bam
NS_WT_APH_S1.sorted.dedup.bam
NS_WT_APH_S2.sorted.dedup.bam
NS_WT_S1.sorted.dedup.bam
NS_WT_S2.sorted.dedup.bam

Aph_MTBP_rep1.sorted.dedup.bam
Aph_MTBP_rep2.sorted.dedup.bam
APH_pRQ4_rep1.sorted.dedup.bam
APH_pRQ4_rep2.sorted.dedup.bam
APH_rec_36hr_MTBP_rep1.sorted.dedup.bam
APH_rec_36hr_MTBP_rep2.sorted.dedup.bam
APH_rec_36hr_pRQ4_rep1.sorted.dedup.bam
APH_rec_36hr_pRQ4_rep2.sorted.dedup.bam
AS_Treslin_rep1.sorted.dedup.bam
AS_Treslin_rep2.sorted.dedup.bam
DTB_0hr_Treslin_rep1.sorted.dedup.bam
DTB_0hr_Treslin_rep2.sorted.dedup.bam
DTB_1hr_Treslin_rep1.sorted.dedup.bam

DTB_1hr_Treslin_rep2.sorted.dedup.bam
DTB_4hr_Treslin_rep1.sorted.dedup.bam
DTB_4hr_Treslin_rep2.sorted.dedup.bam
DTB_8hr_Treslin_rep1.sorted.dedup.bam
DTB_8hr_Treslin_rep2.sorted.dedup.bam
H3Y_Treslin_rep1.sorted.dedup.bam
H3Y_Treslin_rep2.sorted.dedup.bam
Input_36hr.sorted.dedup.bam
Input_Aph.sorted.dedup.bam
Input_Noc_10hr.sorted.dedup.bam
Input_Noc_3hr.sorted.dedup.bam
Input_S139A.sorted.dedup.bam
Input_veh.sorted.dedup.bam
Input_WTM2.sorted.dedup.bam
Noc_10hr_MTBP_rep1.sorted.dedup.bam
Noc_10hr_MTBP_rep2.sorted.dedup.bam
Noc_10hr_Treslin_rep1.sorted.dedup.bam
Noc_10hr_Treslin_rep2.sorted.dedup.bam
Noc_3hr_MTBP_rep1.sorted.dedup.bam
Noc_3hr_MTBP_rep2.sorted.dedup.bam
Noc_3hr_Treslin_rep1.sorted.dedup.bam
Noc_3hr_Treslin_rep2.sorted.dedup.bam
S108A_Ex527_tRQ4_rep1.sorted.dedup.bam
S108A_Ex527_tRQ4_rep2.sorted.dedup.bam
S108A_pS139_Ex527_rep1.sorted.dedup.bam
S108A_pS139_Ex527_rep2.sorted.dedup.bam
S108A_pS139_UT_rep1.sorted.dedup.bam
S108A_pS139_UT_rep2.sorted.dedup.bam
S108A_tRQ4_rep1.sorted.dedup.bam
S108A_tRQ4_rep2.sorted.dedup.bam
S139A_MTBP_rep1.sorted.dedup.bam
S139A_MTBP_rep2.sorted.dedup.bam
S139A_pRQ4_rep1.sorted.dedup.bam
S139A_pRQ4_rep2.sorted.dedup.bam
Veh_MTBP_rep1.sorted.dedup.bam
Veh_MTBP_rep2.sorted.dedup.bam
Veh_pRQ4_rep1.sorted.dedup.bam
Veh_pRQ4_rep2.sorted.dedup.bam
WTM2_Ex527_tRQ4_rep1.sorted.dedup.bam
WTM2_Ex527_tRQ4_rep2.sorted.dedup.bam
WTM2_MTBP_rep1.sorted.dedup.bam
WTM2_MTBP_rep2.sorted.dedup.bam
WTM2_pRQ4_rep1.sorted.dedup.bam
WTM2_pRQ4_rep2.sorted.dedup.bam
WTM2_pS139_Ex527_rep1.sorted.dedup.bam
WTM2_pS139_Ex527_rep2.sorted.dedup.bam
WTM2_pS139_UT_rep1.sorted.dedup.bam
WTM2_pS139_UT_rep2.sorted.dedup.bam
WTM2_tRQ4_rep1.sorted.dedup.bam
WTM2_tRQ4_rep2.sorted.dedup.bam
WT_Treslin_rep1.sorted.dedup.bam
WT_Treslin_rep2.sorted.dedup.bam
Genomic_S1.sorted.dedup.bam
Genomic_S2.sorted.dedup.bam
NS_KORQ4_S1.sorted.dedup.bam
NS_KORQ4_S2.sorted.dedup.bam
NS_S89A_S1.sorted.dedup.bam
NS_S89A_S2.sorted.dedup.bam
NS_WTRQ4_S1.sorted.dedup.bam
NS_WTRQ4_S2.sorted.dedup.bam
RNAseA_cont_S1.sorted.dedup.bam
RNAseA_cont_S2.sorted.dedup.bam

Bigwigs:
APH_rec_1hr_MTBP_rep1.sorted.dedup.bam.scaled.bw
APH_rec_1hr_MTBP_rep2.sorted.dedup.bam.scaled.bw
APH_rec_1hr_pRQ4_rep1.sorted.dedup.bam.scaled.bw
APH_rec_1hr_pRQ4_rep2.sorted.dedup.bam.scaled.bw
APH_rec_4hr_MTBP_rep1.sorted.dedup.bam.scaled.bw
APH_rec_4hr_MTBP_rep2.sorted.dedup.bam.scaled.bw
APH_rec_4hr_pRQ4_rep1.sorted.dedup.bam.scaled.bw
APH_rec_4hr_pRQ4_rep2.sorted.dedup.bam.scaled.bw
APH_rec_8hr_MTBP_rep1.sorted.dedup.bam.scaled.bw

APH_rec_8hr_MTBP_rep2.sorted.dedup.bam.scaled.bw
APH_rec_8hr_pRQ4_rep1.sorted.dedup.bam.scaled.bw
APH_rec_8hr_pRQ4_rep2.sorted.dedup.bam.scaled.bw
AS_MTBP_rep1.sorted.dedup.bam.scaled.bw
AS_MTBP_rep2.sorted.dedup.bam.scaled.bw
AS_pMCM2_rep1.sorted.dedup.bam.scaled.bw
AS_pMCM2_rep2.sorted.dedup.bam.scaled.bw
AS_pRQ4_rep1.sorted.dedup.bam.scaled.bw
AS_pRQ4_rep2.sorted.dedup.bam.scaled.bw
DTB_0hr_MTBP_rep1.sorted.dedup.bam.scaled.bw
DTB_0hr_MTBP_rep2.sorted.dedup.bam.scaled.bw
DTB_0hr_pMCM2_rep1.sorted.dedup.bam.scaled.bw
DTB_0hr_pMCM2_rep2.sorted.dedup.bam.scaled.bw
DTB_0hr_pRQ4_rep1.sorted.dedup.bam.scaled.bw
DTB_0hr_pRQ4_rep2.sorted.dedup.bam.scaled.bw
DTB_1hr_MTBP_rep1.sorted.dedup.bam.scaled.bw
DTB_1hr_MTBP_rep2.sorted.dedup.bam.scaled.bw
DTB_1hr_pMCM2_rep1.sorted.dedup.bam.scaled.bw
DTB_1hr_pMCM2_rep2.sorted.dedup.bam.scaled.bw
DTB_1hr_pRQ4_rep1.sorted.dedup.bam.scaled.bw
DTB_1hr_pRQ4_rep2.sorted.dedup.bam.scaled.bw
DTB_4hr_MTBP_rep1.sorted.dedup.bam.scaled.bw
DTB_4hr_MTBP_rep2.sorted.dedup.bam.scaled.bw
DTB_4hr_pMCM2_rep1.sorted.dedup.bam.scaled.bw
DTB_4hr_pMCM2_rep2.sorted.dedup.bam.scaled.bw
DTB_4hr_pRQ4_rep1.sorted.dedup.bam.scaled.bw
DTB_4hr_pRQ4_rep2.sorted.dedup.bam.scaled.bw
DTB_8hr_MTBP_rep1.sorted.dedup.bam.scaled.bw
DTB_8hr_MTBP_rep2.sorted.dedup.bam.scaled.bw
DTB_8hr_pMCM2_rep1.sorted.dedup.bam.scaled.bw
DTB_8hr_pMCM2_rep2.sorted.dedup.bam.scaled.bw
DTB_8hr_pRQ4_rep1.sorted.dedup.bam.scaled.bw
DTB_8hr_pRQ4_rep2.sorted.dedup.bam.scaled.bw
DTB_Input_1hr_R1_001.sorted.dedup.bam.scaled.bw
DTB_Input_4hr_R1_001.sorted.dedup.bam.scaled.bw
DTB_Input_8hr_R1_001.sorted.dedup.bam.scaled.bw
H3Y_MTBP_rep1.sorted.dedup.bam.scaled.bw
H3Y_MTBP_rep2.sorted.dedup.bam.scaled.bw
H3Y_pMCM2_rep1.sorted.dedup.bam.scaled.bw
H3Y_pMCM2_rep2.sorted.dedup.bam.scaled.bw
H3Y_pRQ4_rep1.sorted.dedup.bam.scaled.bw
H3Y_pRQ4_rep2.sorted.dedup.bam.scaled.bw
H3Y_RQ4_rep1.sorted.dedup.bam.scaled.bw
H3Y_RQ4_rep2.sorted.dedup.bam.scaled.bw
Input_AS_R1_001.sorted.dedup.bam.scaled.bw
Input_H3Y_R1_001.sorted.dedup.bam.scaled.bw
Input_S89A_RQ_R1_001.sorted.dedup.bam.scaled.bw
Input_UT_1_R1_001.sorted.dedup.bam.scaled.bw
Input_UT_2_R1_001.sorted.dedup.bam.scaled.bw
Input_WT_APH_R1_001.sorted.dedup.bam.scaled.bw
Input_WT_M2.sorted.dedup.bam.scaled.bw
Input_WT_R1_001.sorted.dedup.bam.scaled.bw
Input_WT_RQ_R1_001.sorted.dedup.bam.scaled.bw
MCM2_WT_Ex_MTBP_rep1.sorted.dedup.bam.scaled.bw
MCM2_WT_Ex_MTBP_rep2.sorted.dedup.bam.scaled.bw
MCM2_WT_Ex_pRQ4_rep1.sorted.dedup.bam.scaled.bw
MCM2_WT_Ex_pRQ4_rep2.sorted.dedup.bam.scaled.bw
MCM2_WT_MTBP_rep1.sorted.dedup.bam.scaled.bw
MCM2_WT_MTBP_rep2.sorted.dedup.bam.scaled.bw
MCM2_WT_pRQ4_rep1.sorted.dedup.bam.scaled.bw
MCM2_WT_pRQ4_rep2.sorted.dedup.bam.scaled.bw
RQ4_null_MTBP_APH_rep1.sorted.dedup.bam.scaled.bw
RQ4_null_MTBP_APH_rep2.sorted.dedup.bam.scaled.bw
RQ4_null_MTBP_UT_rep1.sorted.dedup.bam.scaled.bw
RQ4_null_MTBP_UT_rep2.sorted.dedup.bam.scaled.bw
RQ4_null_pRQ4_APH_rep1.sorted.dedup.bam.scaled.bw
RQ4_null_pRQ4_APH_rep2.sorted.dedup.bam.scaled.bw
RQ4_null_pRQ4_UT_rep1.sorted.dedup.bam.scaled.bw
RQ4_null_pRQ4_UT_rep2.sorted.dedup.bam.scaled.bw
RQ4_WT_MTBP_APH_rep1.sorted.dedup.bam.scaled.bw
RQ4_WT_MTBP_APH_rep2.sorted.dedup.bam.scaled.bw
RQ4_WT_MTBP_UT_rep1.sorted.dedup.bam.scaled.bw
RQ4_WT_MTBP_UT_rep2.sorted.dedup.bam.scaled.bw
RQ4_WT_pRQ4_APH_rep1.sorted.dedup.bam.scaled.bw
RQ4_WT_pRQ4_APH_rep2.sorted.dedup.bam.scaled.bw
RQ4_WT_pRQ4_UT_rep1.sorted.dedup.bam.scaled.bw

RQ4_WT_pRQ4_UT_rep2.sorted.dedup.bam.scaled.bw
S108A_Ex_MTBP_rep1.sorted.dedup.bam.scaled.bw
S108A_Ex_MTBP_rep2.sorted.dedup.bam.scaled.bw
S108A_Ex_pRQ4_rep1.sorted.dedup.bam.scaled.bw
S108A_Ex_pRQ4_rep2.sorted.dedup.bam.scaled.bw
S108A_MTBP_rep1.sorted.dedup.bam.scaled.bw
S108A_MTBP_rep2.sorted.dedup.bam.scaled.bw
S108A_pRQ4_rep1.sorted.dedup.bam.scaled.bw
S108A_pRQ4_rep2.sorted.dedup.bam.scaled.bw
S89A_MTBP_APH_rep1.sorted.dedup.bam.scaled.bw
S89A_MTBP_APH_rep2.sorted.dedup.bam.scaled.bw
S89A_MTBP_UT_rep1.sorted.dedup.bam.scaled.bw
S89A_MTBP_UT_rep2.sorted.dedup.bam.scaled.bw
S89A_pRQ4_APH_rep1.sorted.dedup.bam.scaled.bw
S89A_pRQ4_APH_rep2.sorted.dedup.bam.scaled.bw
S89A_pRQ4_UT_rep1.sorted.dedup.bam.scaled.bw
S89A_pRQ4_UT_rep2.sorted.dedup.bam.scaled.bw
UT_MTBP_rep1.sorted.dedup.bam.scaled.bw
UT_MTBP_rep2.sorted.dedup.bam.scaled.bw
UT_pRQ4_rep1.sorted.dedup.bam.scaled.bw
UT_pRQ4_rep2.sorted.dedup.bam.scaled.bw
WT_APH_MTBP_rep1.sorted.dedup.bam.scaled.bw
WT_APH_MTBP_rep2.sorted.dedup.bam.scaled.bw
WT_APH_pMCM2_rep1.sorted.dedup.bam.scaled.bw
WT_APH_pMCM2_rep2.sorted.dedup.bam.scaled.bw
WT_APH_pRQ4_rep1.sorted.dedup.bam.scaled.bw
WT_APH_pRQ4_rep2.sorted.dedup.bam.scaled.bw
WT_APH_RQ4_rep1.sorted.dedup.bam.scaled.bw
WT_APH_RQ4_rep2.sorted.dedup.bam.scaled.bw
WT_MTBP_rep1.sorted.dedup.bam.scaled.bw
WT_MTBP_rep2.sorted.dedup.bam.scaled.bw
WT_pMCM2_rep1.sorted.dedup.bam.scaled.bw
WT_pMCM2_rep2.sorted.dedup.bam.scaled.bw
WT_pRQ4_rep1.sorted.dedup.bam.scaled.bw
WT_pRQ4_rep2.sorted.dedup.bam.scaled.bw
WT_RQ4_rep1.sorted.dedup.bam.scaled.bw
WT_RQ4_rep2.sorted.dedup.bam.scaled.bw
NS_H3Y_S1.sorted.dedup.bam.scaled.bw
NS_H3Y_S2.sorted.dedup.bam.scaled.bw
NS_WT_0hr_S1.sorted.dedup.bam.scaled.bw
NS_WT_0hr_S2.sorted.dedup.bam.scaled.bw
NS_WT_1hr_S1.sorted.dedup.bam.scaled.bw
NS_WT_1hr_S2.sorted.dedup.bam.scaled.bw
NS_WT_4hr_S1.sorted.dedup.bam.scaled.bw
NS_WT_4hr_S2.sorted.dedup.bam.scaled.bw
NS_WT_7hr_S1.sorted.dedup.bam.scaled.bw
NS_WT_7hr_S2.sorted.dedup.bam.scaled.bw
NS_WT_APH_S1.sorted.dedup.bam.scaled.bw
NS_WT_APH_S2.sorted.dedup.bam.scaled.bw
NS_WT_S1.sorted.dedup.bam.scaled.bw
NS_WT_S2.sorted.dedup.bam.scaled.bw

Aph_MTBP_rep1.sorted.dedup.bam.scaled.bw
Aph_MTBP_rep2.sorted.dedup.bam.scaled.bw
APH_pRQ4_rep1.sorted.dedup.bam.scaled.bw
APH_pRQ4_rep2.sorted.dedup.bam.scaled.bw
APH_rec_36hr_MTBP_rep1.sorted.dedup.bam.scaled.bw
APH_rec_36hr_MTBP_rep2.sorted.dedup.bam.scaled.bw
APH_rec_36hr_pRQ4_rep1.sorted.dedup.bam.scaled.bw
APH_rec_36hr_pRQ4_rep2.sorted.dedup.bam.scaled.bw
AS_Treslin_rep1.sorted.dedup.bam.scaled.bw
AS_Treslin_rep2.sorted.dedup.bam.scaled.bw
DTB_0hr_Treslin_rep1.sorted.dedup.bam.scaled.bw
DTB_0hr_Treslin_rep2.sorted.dedup.bam.scaled.bw
DTB_1hr_Treslin_rep1.sorted.dedup.bam.scaled.bw
DTB_1hr_Treslin_rep2.sorted.dedup.bam.scaled.bw
DTB_4hr_Treslin_rep1.sorted.dedup.bam.scaled.bw
DTB_4hr_Treslin_rep2.sorted.dedup.bam.scaled.bw
DTB_8hr_Treslin_rep1.sorted.dedup.bam.scaled.bw
DTB_8hr_Treslin_rep2.sorted.dedup.bam.scaled.bw
H3Y_Treslin_rep1.sorted.dedup.bam.scaled.bw
H3Y_Treslin_rep2.sorted.dedup.bam.scaled.bw
Input_36hr.sorted.dedup.bam.scaled.bw
Input_Aph.sorted.dedup.bam.scaled.bw
Input_Noc_10hr.sorted.dedup.bam.scaled.bw
Input_Noc_3hr.sorted.dedup.bam.scaled.bw

Input_S139A.sorted.dedup.bam.scaled.bw
Input_veh.sorted.dedup.bam.scaled.bw
Input_WTM2.sorted.dedup.bam.scaled.bw
Noc_10hr_MTBP_rep1.sorted.dedup.bam.scaled.bw
Noc_10hr_MTBP_rep2.sorted.dedup.bam.scaled.bw
Noc_10hr_Treslin_rep1.sorted.dedup.bam.scaled.bw
Noc_10hr_Treslin_rep2.sorted.dedup.bam.scaled.bw
Noc_3hr_MTBP_rep1.sorted.dedup.bam.scaled.bw
Noc_3hr_MTBP_rep2.sorted.dedup.bam.scaled.bw
Noc_3hr_Treslin_rep1.sorted.dedup.bam.scaled.bw
Noc_3hr_Treslin_rep2.sorted.dedup.bam.scaled.bw
S108A_Ex527_tRQ4_rep1.sorted.dedup.bam.scaled.bw
S108A_Ex527_tRQ4_rep2.sorted.dedup.bam.scaled.bw
S108A_pS139_Ex527_rep1.sorted.dedup.bam.scaled.bw
S108A_pS139_Ex527_rep2.sorted.dedup.bam.scaled.bw
S108A_pS139_UT_rep1.sorted.dedup.bam.scaled.bw
S108A_pS139_UT_rep2.sorted.dedup.bam.scaled.bw
S108A_tRQ4_rep1.sorted.dedup.bam.scaled.bw
S108A_tRQ4_rep2.sorted.dedup.bam.scaled.bw
S139A_MTBP_rep1.sorted.dedup.bam.scaled.bw
S139A_MTBP_rep2.sorted.dedup.bam.scaled.bw
S139A_pRQ4_rep1.sorted.dedup.bam.scaled.bw
S139A_pRQ4_rep2.sorted.dedup.bam.scaled.bw
Veh_MTBP_rep1.sorted.dedup.bam.scaled.bw
Veh_MTBP_rep2.sorted.dedup.bam.scaled.bw
Veh_pRQ4_rep1.sorted.dedup.bam.scaled.bw
Veh_pRQ4_rep2.sorted.dedup.bam.scaled.bw
WTM2_Ex527_tRQ4_rep1.sorted.dedup.bam.scaled.bw
WTM2_Ex527_tRQ4_rep2.sorted.dedup.bam.scaled.bw
WTM2_MTBP_rep1.sorted.dedup.bam.scaled.bw
WTM2_MTBP_rep2.sorted.dedup.bam.scaled.bw
WTM2_pRQ4_rep1.sorted.dedup.bam.scaled.bw
WTM2_pRQ4_rep2.sorted.dedup.bam.scaled.bw
WTM2_pS139_Ex527_rep1.sorted.dedup.bam.scaled.bw
WTM2_pS139_Ex527_rep2.sorted.dedup.bam.scaled.bw
WTM2_pS139_UT_rep1.sorted.dedup.bam.scaled.bw
WTM2_pS139_UT_rep2.sorted.dedup.bam.scaled.bw
WTM2_tRQ4_rep1.sorted.dedup.bam.scaled.bw
WTM2_tRQ4_rep2.sorted.dedup.bam.scaled.bw
WT_Treslin_rep1.sorted.dedup.bam.scaled.bw
WT_Treslin_rep2.sorted.dedup.bam.scaled.bw
Genomic_S1.sorted.dedup.bam.scaled.bw
Genomic_S2.sorted.dedup.bam.scaled.bw
NS_KORQ4_S1.sorted.dedup.bam.scaled.bw
NS_KORQ4_S2.sorted.dedup.bam.scaled.bw
NS_S89A_S1.sorted.dedup.bam.scaled.bw
NS_S89A_S2.sorted.dedup.bam.scaled.bw
NS_WTRQ4_S1.sorted.dedup.bam.scaled.bw
NS_WTRQ4_S2.sorted.dedup.bam.scaled.bw
RNAseA_cont_S1.sorted.dedup.bam.scaled.bw
RNAseA_cont_S2.sorted.dedup.bam.scaled.bw

Peak files :
Aph_MTBP_rep1_peaks.narrowPeak
Aph_MTBP_rep2_peaks.narrowPeak
APH_pRQ4_rep1_peaks.narrowPeak
APH_pRQ4_rep2_peaks.narrowPeak
APH_rec_36hr_MTBP_rep1_peaks.narrowPeak
APH_rec_36hr_MTBP_rep2_peaks.narrowPeak
APH_rec_36hr_pRQ4_rep1_peaks.narrowPeak
APH_rec_36hr_pRQ4_rep2_peaks.narrowPeak
AS_Treslin_rep1_peaks.narrowPeak
AS_Treslin_rep2_peaks.narrowPeak
DTB_0hr_Treslin_rep1_peaks.narrowPeak
DTB_0hr_Treslin_rep2_peaks.narrowPeak
DTB_1hr_Treslin_rep1_peaks.narrowPeak
DTB_1hr_Treslin_rep2_peaks.narrowPeak
DTB_4hr_Treslin_rep1_peaks.narrowPeak
DTB_4hr_Treslin_rep2_peaks.narrowPeak
DTB_8hr_Treslin_rep1_peaks.narrowPeak
DTB_8hr_Treslin_rep2_peaks.narrowPeak
H3Y_Treslin_rep1_peaks.narrowPeak
H3Y_Treslin_rep2_peaks.narrowPeak

Input_36hr_peaks.narrowPeak
Input_Aph_peaks.narrowPeak
Input_Noc_10hr_peaks.narrowPeak
Input_Noc_3hr_peaks.narrowPeak
Input_S139A_peaks.narrowPeak
Input_veh_peaks.narrowPeak
Input_WTM2_peaks.narrowPeak
Noc_10hr_MTBP_rep1_peaks.narrowPeak
Noc_10hr_MTBP_rep2_peaks.narrowPeak
Noc_10hr_Treslin_rep1_peaks.narrowPeak
Noc_10hr_Treslin_rep2_peaks.narrowPeak
Noc_3hr_MTBP_rep1_peaks.narrowPeak
Noc_3hr_MTBP_rep2_peaks.narrowPeak
Noc_3hr_Treslin_rep1_peaks.narrowPeak
Noc_3hr_Treslin_rep2_peaks.narrowPeak
S108A_Ex527_tRQ4_rep1_peaks.narrowPeak
S108A_Ex527_tRQ4_rep2_peaks.narrowPeak
S108A_pS139_Ex527_rep1_peaks.narrowPeak
S108A_pS139_Ex527_rep2_peaks.narrowPeak
S108A_pS139_UT_rep1_peaks.narrowPeak
S108A_pS139_UT_rep2_peaks.narrowPeak
S108A_tRQ4_rep1_peaks.narrowPeak
S108A_tRQ4_rep2_peaks.narrowPeak
S139A_MTBP_rep1_peaks.narrowPeak
S139A_MTBP_rep2_peaks.narrowPeak
S139A_pRQ4_rep1_peaks.narrowPeak
S139A_pRQ4_rep2_peaks.narrowPeak
Veh_MTBP_rep1_peaks.narrowPeak
Veh_MTBP_rep2_peaks.narrowPeak
Veh_pRQ4_rep1_peaks.narrowPeak
Veh_pRQ4_rep2_peaks.narrowPeak
WTM2_Ex527_tRQ4_rep1_peaks.narrowPeak
WTM2_Ex527_tRQ4_rep2_peaks.narrowPeak
WTM2_MTBP_rep1_peaks.narrowPeak
WTM2_MTBP_rep2_peaks.narrowPeak
WTM2_pRQ4_rep1_peaks.narrowPeak
WTM2_pRQ4_rep2_peaks.narrowPeak
WTM2_pS139_Ex527_rep1_peaks.narrowPeak
WTM2_pS139_Ex527_rep2_peaks.narrowPeak
WTM2_pS139_UT_rep1_peaks.narrowPeak
WTM2_pS139_UT_rep2_peaks.narrowPeak
WTM2_tRQ4_rep1_peaks.narrowPeak
WTM2_tRQ4_rep2_peaks.narrowPeak
WT_Treslin_rep1_peaks.narrowPeak
WT_Treslin_rep2_peaks.narrowPeak
Genomic_S1_peaks.broadPeak
Genomic_S2_peaks.broadPeak
NS_KORQ4_S1_peaks.broadPeak
NS_KORQ4_S2_peaks.broadPeak
NS_S89A_S1_peaks.broadPeak
NS_S89A_S2_peaks.broadPeak
NS_WTRQ4_S1_peaks.broadPeak
NS_WTRQ4_S2_peaks.broadPeak
RNAseA_cont_S1_peaks.broadPeak
RNAseA_cont_S2_peaks.broadPeak

APH_rec_1hr_MTBP_rep1_peaks.narrowPeak
APH_rec_1hr_MTBP_rep2_peaks.narrowPeak
APH_rec_1hr_pRQ4_rep1_peaks.narrowPeak
APH_rec_1hr_pRQ4_rep2_peaks.narrowPeak
APH_rec_4hr_MTBP_rep1_peaks.narrowPeak
APH_rec_4hr_MTBP_rep2_peaks.narrowPeak
APH_rec_4hr_pRQ4_rep1_peaks.narrowPeak
APH_rec_4hr_pRQ4_rep2_peaks.narrowPeak
APH_rec_8hr_MTBP_rep1_peaks.narrowPeak
APH_rec_8hr_MTBP_rep2_peaks.narrowPeak
APH_rec_8hr_pRQ4_rep1_peaks.narrowPeak
APH_rec_8hr_pRQ4_rep2_peaks.narrowPeak
AS_MTBP_rep1_peaks.narrowPeak
AS_MTBP_rep2_peaks.narrowPeak
AS_pMCM2_rep1_peaks.narrowPeak
AS_pMCM2_rep2_peaks.narrowPeak
AS_pRQ4_rep1_peaks.narrowPeak
AS_pRQ4_rep2_peaks.narrowPeak
DTB_0hr_MTBP_rep1_peaks.narrowPeak
DTB_0hr_MTBP_rep2_peaks.narrowPeak

DTB_0hr_pMCM2_rep1_peaks.narrowPeak
DTB_0hr_pMCM2_rep2_peaks.narrowPeak
DTB_0hr_pRQ4_rep1_peaks.narrowPeak
DTB_0hr_pRQ4_rep2_peaks.narrowPeak
DTB_1hr_MTBP_rep1_peaks.narrowPeak
DTB_1hr_MTBP_rep2_peaks.narrowPeak
DTB_1hr_pMCM2_rep1_peaks.narrowPeak
DTB_1hr_pMCM2_rep2_peaks.narrowPeak
DTB_1hr_pRQ4_rep1_peaks.narrowPeak
DTB_1hr_pRQ4_rep2_peaks.narrowPeak
DTB_4hr_MTBP_rep1_peaks.narrowPeak
DTB_4hr_MTBP_rep2_peaks.narrowPeak
DTB_4hr_pMCM2_rep1_peaks.narrowPeak
DTB_4hr_pMCM2_rep2_peaks.narrowPeak
DTB_4hr_pRQ4_rep1_peaks.narrowPeak
DTB_4hr_pRQ4_rep2_peaks.narrowPeak
DTB_8hr_MTBP_rep1_peaks.narrowPeak
DTB_8hr_MTBP_rep2_peaks.narrowPeak
DTB_8hr_pMCM2_rep1_peaks.narrowPeak
DTB_8hr_pMCM2_rep2_peaks.narrowPeak
DTB_8hr_pRQ4_rep1_peaks.narrowPeak
DTB_8hr_pRQ4_rep2_peaks.narrowPeak
DTB_Input_1hr_R1_001_peaks.narrowPeak
DTB_Input_4hr_R1_001_peaks.narrowPeak
DTB_Input_8hr_R1_001_peaks.narrowPeak
H3Y_MTBP_rep1_peaks.narrowPeak
H3Y_MTBP_rep2_peaks.narrowPeak
H3Y_pMCM2_rep1_peaks.narrowPeak
H3Y_pMCM2_rep2_peaks.narrowPeak
H3Y_pRQ4_rep1_peaks.narrowPeak
H3Y_pRQ4_rep2_peaks.narrowPeak
H3Y_RQ4_rep1_peaks.narrowPeak
H3Y_RQ4_rep2_peaks.narrowPeak
Input_AS_R1_001_peaks.narrowPeak
Input_H3Y_R1_001_peaks.narrowPeak
Input_S89A_RQ_R1_001_peaks.narrowPeak
Input_UT_1_R1_001_peaks.narrowPeak
Input_UT_2_R1_001_peaks.narrowPeak
Input_WT_APH_R1_001_peaks.narrowPeak
Input_WT_M2_peaks.narrowPeak

Input_WT_RQ_R1_001_peaks.narrowPeak
MCM2_WT_Ex_MTBP_rep1_peaks.narrowPeak
MCM2_WT_Ex_MTBP_rep2_peaks.narrowPeak
MCM2_WT_Ex_pRQ4_rep1_peaks.narrowPeak
MCM2_WT_Ex_pRQ4_rep2_peaks.narrowPeak
MCM2_WT_MTBP_rep1_peaks.narrowPeak
MCM2_WT_MTBP_rep2_peaks.narrowPeak
MCM2_WT_pRQ4_rep1_peaks.narrowPeak
MCM2_WT_pRQ4_rep2_peaks.narrowPeak
RQ4_null_MTBP_APH_rep1_peaks.narrowPeak
RQ4_null_MTBP_APH_rep2_peaks.narrowPeak
RQ4_null_MTBP_UT_rep1_peaks.narrowPeak
RQ4_null_MTBP_UT_rep2_peaks.narrowPeak
RQ4_null_pRQ4_APH_rep1_peaks.narrowPeak
RQ4_null_pRQ4_APH_rep2_peaks.narrowPeak
RQ4_null_pRQ4_UT_rep1_peaks.narrowPeak
RQ4_null_pRQ4_UT_rep2_peaks.narrowPeak
RQ4_WT_MTBP_APH_rep1_peaks.narrowPeak
RQ4_WT_MTBP_APH_rep2_peaks.narrowPeak
RQ4_WT_MTBP_UT_rep1_peaks.narrowPeak
RQ4_WT_MTBP_UT_rep2_peaks.narrowPeak
RQ4_WT_pRQ4_APH_rep1_peaks.narrowPeak
RQ4_WT_pRQ4_APH_rep2_peaks.narrowPeak
RQ4_WT_pRQ4_UT_rep1_peaks.narrowPeak
RQ4_WT_pRQ4_UT_rep2_peaks.narrowPeak
S108A_Ex_MTBP_rep1_peaks.narrowPeak
S108A_Ex_MTBP_rep2_peaks.narrowPeak
S108A_Ex_pRQ4_rep1_peaks.narrowPeak
S108A_Ex_pRQ4_rep2_peaks.narrowPeak
S108A_MTBP_rep1_peaks.narrowPeak
S108A_MTBP_rep2_peaks.narrowPeak
S108A_pRQ4_rep1_peaks.narrowPeak
S108A_pRQ4_rep2_peaks.narrowPeak
S89A_MTBP_APH_rep1_peaks.narrowPeak
S89A_MTBP_APH_rep2_peaks.narrowPeak

S89A_MTBP_UT_rep1_peaks.narrowPeak
S89A_MTBP_UT_rep2_peaks.narrowPeak
S89A_pRQ4_APH_rep1_peaks.narrowPeak
S89A_pRQ4_APH_rep2_peaks.narrowPeak
S89A_pRQ4_UT_rep1_peaks.narrowPeak
S89A_pRQ4_UT_rep2_peaks.narrowPeak
UT_MTBP_rep1_peaks.narrowPeak
UT_MTBP_rep2_peaks.narrowPeak
UT_pRQ4_rep1_peaks.narrowPeak
UT_pRQ4_rep2_peaks.narrowPeak
WT_APH_MTBP_rep1_peaks.narrowPeak
WT_APH_MTBP_rep2_peaks.narrowPeak
WT_APH_pMCM2_rep1_peaks.narrowPeak
WT_APH_pMCM2_rep2_peaks.narrowPeak
WT_APH_pRQ4_rep1_peaks.narrowPeak
WT_APH_pRQ4_rep2_peaks.narrowPeak
WT_APH_RQ4_rep1_peaks.narrowPeak
WT_APH_RQ4_rep2_peaks.narrowPeak
WT_MTBP_rep1_peaks.narrowPeak
WT_MTBP_rep2_peaks.narrowPeak
WT_pMCM2_rep1_peaks.narrowPeak
WT_pMCM2_rep2_peaks.narrowPeak
WT_pRQ4_rep1_peaks.narrowPeak
WT_pRQ4_rep2_peaks.narrowPeak
WT_RQ4_rep1_peaks.narrowPeak
WT_RQ4_rep2_peaks.narrowPeak
NS_H3Y_S1_peaks.broadPeak
NS_H3Y_S2_peaks.broadPeak
NS_WT_0hr_S1_peaks.broadPeak
NS_WT_0hr_S2_peaks.broadPeak
NS_WT_1hr_S1_peaks.broadPeak
NS_WT_1hr_S2_peaks.broadPeak
NS_WT_4hr_S1_peaks.broadPeak
NS_WT_4hr_S2_peaks.broadPeak
NS_WT_7hr_S1_peaks.broadPeak
NS_WT_7hr_S2_peaks.broadPeak
NS_WT_APH_S1_peaks.broadPeak
NS_WT_APH_S2_peaks.broadPeak
NS_WT_S1_peaks.broadPeak
NS_WT_S2_peaks.broadPeak

Genome browser session (e.g. UCSC): NA

Methodology

Replicates: 2 biological replicates

Sequencing depth: At least 25 million reads for ChIP-seq and 60 million reads for NS-seq. For all alignment files more than 95 % of reads were mapped.

Antibodies: RecQL4 (Cell Signaling, 2814), phospho-MCM2 (S139) (Cell Signaling, 12958), MTBP (Novus biologicals, NBP1-86408), Treslin (ThermoFisher PA583839); pRecQL4 S89 (this work, see fig S1F for validation)

Peak calling parameters: Peaks with high read coverages were identified by the narrow MACS2 (version 2.1.1.20160309) peak calling method using input DNA as controls. Peaks were filtered using the "peak-score" MACS2 metric in R (version 3.5.1) by accepting regions above the inflection point threshold of "peak-scores" from the raw output.

Data quality: Peak calling was performed with MACS, all the peaks were above the FDR threshold of 0.05.

Software: Trimmomatic (version 0.36) and Trim Galore (version 0.4.5); FastQC (version 0.11.5) [https://www.bioinformatics.babraham.ac.uk/projects/fastqc/], bwa aligner (version 0.7.17);MACS2 (version 2.1.1.20160309); MACS2 metric in R (version 3.5.1); Created R scripts (https://github.com/ncbi/BAMscale/wiki).

Flow Cytometry

Plots

Confirm that:

☒ The axis labels state the marker and fluorochrome used (e.g. CD4-FITC).

☒ The axis scales are clearly visible. Include numbers along axes only for bottom left plot of group (a 'group' is an analysis of identical markers).

☒ All plots are contour plots with outliers or pseudocolor plots.

☒ A numerical value for number of cells or percentage (with statistics) is provided.

Methodology

Sample preparation	Cancer cell lines were processed according to the EdU kit.
Instrument	BD LSR Fortessa cell analyzer.
Software	FACSDiva software for collecting samples and Flowjo 10.6. for analysis.
Cell population abundance	Since we have background information for the parameter detected, antibodies are very good, there are always both negative and positive populations in the same sample, it's pretty straightforward to gate. Since it's pretty straightforward, to avoid too crowd graph, we did not induce axis scales for all the graphs.
Gating strategy	Single cells gated according to DAPI-H DAPI-A were analyzed as the gates shown on each graphs (e.g. gating shown in the source data file).

☒ Tick this box to confirm that a figure exemplifying the gating strategy is provided in the Supplementary Information.
